# Systematic Early Intervention for Bereaved: Study Protocol of a Pilot Randomized Controlled Trial With Families Who Suddenly Lose a Partner and a Parent

**DOI:** 10.2196/resprot.5765

**Published:** 2016-08-03

**Authors:** Mariana Pereira, Kari Dyregrov, May Aa Hauken, Mette Senneseth, Atle Dyregrov

**Affiliations:** ^1^ Center for Crisis Psychology Bergen Norway; ^2^ Faculty of Health and Social Sciences Bergen University College Bergen Norway; ^3^ Department of Clinical Psychology Faculty of Psychology University of Bergen Bergen Norway

**Keywords:** traumatic death, complicated grief, mental health, family functioning, early intervention, randomized controlled trial

## Abstract

**Background:**

Grief has been associated with several long-term negative outcomes for both surviving parents and bereaved children, especially when it is preceded by unnatural and violent deaths. Nevertheless, it has been an underestimated public health problem with few, if any, empirically documented early preventive intervention programs. The best time to start them is also a major question that requires further evidence.

**Objective:**

The overall aim of this study is to assess the feasibility of a future larger trial, informing sample size calculation, recruitment/randomization procedures, retention rates, data collection forms, and outcomes. This study will also explore: (1) the early effects of Systematic Early Intervention for Bereaved (SEIB) compared with the early effects of care as usual, and (2) the effects of the immediate SEIB version compared with the effects of the delayed SEIB version.

**Methods:**

In a pilot randomized controlled trial (RCT) with a delayed intervention design, suddenly bereaved families will be assigned to: the immediate-SEIB intervention group, or the delayed-SEIB intervention group. Participants will fill in a set of self-report measures at baseline, and after 3, 6, and 9 months follow-up. Quantitative data on traumatic stress symptoms, complicated grief, psychological wellbeing, daily functioning, social support, parental capacity, parenting practices, and family functioning will be collected to inform power calculations and explore SEIB’s preliminary effects. Data on the flow of participants throughout the trial will be analyzed in order to estimate recruitment and retention rates. Two brief questionnaires were developed to assess recruitment procedures, randomization, and data collection materials.

**Results:**

Recruitment for this project started in August 2015, and follow-up data collection will be completed in June 2017.

**Conclusions:**

This study prepares the ground work for the design and implementation of a main trial and may add preliminary knowledge to the significance of early supportive practices that have been commonly used regardless of their sparse evidence.

## Introduction

Bereavement is a natural and common event, and most people are generally able to adapt to the resulting grief over time and regain function in their everyday life [1]. However, approximately 10% to 22% of bereaved people experience deleterious forms of mental distress that result in mental health deterioration [2]. The loss of a significantly loved person through death can be a complex and disturbing life event that is linked to greater psychological problems, physical illness, and mortality. Individual grief reactions can vary from minor and shorter responses to more severe and prolonged manifestations [3]. More importantly, unnatural and violent deaths significantly cause more severe after effects than natural deaths [4].

Complicated grief encloses the more severe and prolonged symptoms that hamper social and occupational functioning [3,5]. It has been found to be related to higher rates of mental disorders, sleep problems, suicidality, cardiovascular and cancer diseases, and lack of social support [6-10]. Preoccupied thoughts of the deceased, intense searching and yearning for the deceased, avoiding memories of the deceased, death denying, and crying are among the core symptomatology of complicated grief [11]. In addition, other comorbid symptoms that meet the criteria for depression and/or posttraumatic stress disorder (PTSD) have been documented [12].

Acknowledging the individual and contextual risk factors for the potentially chronic complicated grief outcomes increase awareness on the subset of bereaved people who are in major need of assistance [13]. In several studies, the death of a parent is emphasized as one of the most demanding and traumatic events that can be experienced in childhood and youth [14,15]. It has been found to enhance the risk for a wide range of mental and behavioral problems, even when controlling for previous risk factors [16]. Parentally bereaved children have shown clinically significant indicators of psychological distress (eg, depression, anxiety, somatic complaints, withdrawal), traumatic grief (eg, yearning for the deceased, diminished acceptance of the death), lower academic functioning and self-esteem/self-efficacy, higher external locus of control, and social problems [17-19]. Furthermore, parentally bereaved children who suddenly and unexpectedly lose a parent (eg, following a suicide, an accident, or a natural death) were at greater risk of developing depression and PTSD symptoms [20]. A sudden and unexpected loss is seen as an existential crisis that threatens self-beliefs about safety [21] and self-ability to accept, confront, and adapt to what has occurred [22].

The manifold negative effects of parental death in childhood seem to be linked to increased rates of disorder in both bereaved parents and their children [20,23]. The surviving parents have to raise their children under extremely difficult conditions. On the one hand, they must deal with the loss of their partner and their own psychological problems, while facing the pressure of being a single parent [24]. On the other hand, parentally bereaved children can pose additional challenges for parents, expressing their adjustment problems through more disruptive behaviors [25].

In view of these detrimental influences of bereavement-related psychological distress on both the individual and the family system, it is crucial to break this cascade of negative cycles. It highlights the need of developing effective interventions aimed to promote resilience for both bereaved parents and their children, especially in the early stages that follow death [26,27] where the disabling consequences have been more strongly noticed [3]. Several clinicians and researchers in the loss and trauma field have emphasized the usefulness of early crisis intervention [26-28]. They argue that early intervention favors the attenuation of the initial dysfunctional appraisals and enables a better case management, and maximizes the chances of a more adaptive developmental pathway [27]. Norwegian studies on the users’ perspectives show that traumatic bereaved participants ask for: (1) immediate assistance, (2) outreach help, (3) help for their children, (4) information about the event and potential reactions, (5) possibility to meet with others who experienced similar situations, and (6) help over time [22,29]. Nevertheless, meta-analytic findings failed to show a significant effect of preventive approaches, pinpointing diverse methodological limitations among the studies [30]. To our knowledge, none of these studies assesses interventions taking place immediately after the loss. The start of these preventive programs ranges from 2 to 6 months post loss [31,32]. Thus, it is essential to develop well-designed randomized controlled studies [33] attempting to increase understanding about the best time to provide early intervention.

In line with this, the Systematic Early Intervention for Bereaved (SEIB) was developed. It is the first Norwegian program that proposes professional family assistance starting in the first days after a sudden and traumatic loss. Within a family perspective, SEIB seeks to facilitate natural mourning, resolve grief complications, promote parental capacity, and help the bereaved partner and their children to develop or enhance satisfying relations/activities, and to regain control over their life. The basis of this approach is from the field of early crisis intervention [26-28]. The existing literature [3,25], the wishes for help as expressed by the bereaved themselves [22,29], and our extensive clinical practice, are at the heart of this approach.

The present study aims to expand knowledge in early intervention on family -related psychological distress following a sudden and unexpected death of a partner/parent. It consists of a pilot study to assess the feasibility of a future main trial, as well as SEIB’s preliminary impact with suddenly bereaved families. Accordingly, the specific aims are to:

1. Assess recruitment materials and procedures.

2. Assess the usefulness of randomization and data collection materials.

3. Obtain reliable estimates regarding recruitment and retention.

4. Provide information on power calculations and possible outcomes (both parents and children).

5. Explore the 3-month SEIB effects' on traumatic stress symptoms, complicated grief symptoms, psychological wellbeing, daily functioning, social support, parental capacity, parenting practices, and family functioning, compared with the 3-month effects of care as usual.

6. Explore the 3- and 6-month effects of the immediate-SEIB version on traumatic stress symptoms, complicated grief symptoms, psychological wellbeing, daily functioning, social support, parental capacity, parenting practices, and family functioning, compared with the 6- and 9-month effects of the delayed-SEIB version.

## Methods

### Trial Design

**Figure 1:**
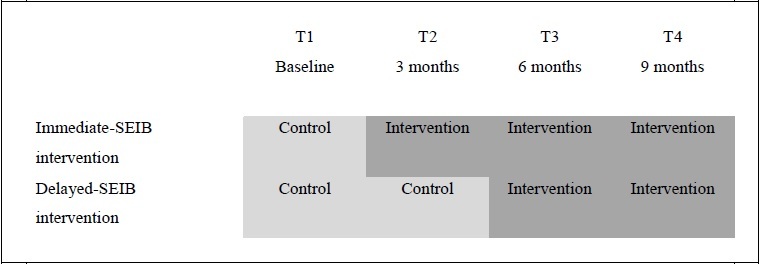
Design of the study protocol.

As outlined in Figure 1, this study is a one-center, pilot, randomized controlled trial (RCT) with a delayed intervention design in which suddenly bereaved families will be randomly assigned to: the immediate-SEIB intervention group (receiving SEIB in the aftermath of the loss) or the delayed-SEIB intervention group (control condition; receiving SEIB 3 months post loss). Both groups can make use of care as usual from their communities.

### Participants and Eligibility Criteria

The sample will include bereaved families with children younger than 18 -years old, who suddenly and unexpectedly lose a partner and a parent. Bereaved partners and their children, aged from 12 - to 18 -years old, will be the informants of the study. To ensure a homogeneous sample, all family members must speak Norwegian. Eligible families are those who satisfy both of the criteria listed in Textbox 1.

**Inclusion criteria.**

- Criterion for unexpectedness: the loss shall occur following an accident, a suicide, a murder, or a disease, as well as situations where people are missing (presumed dead).
- Criterion for suddenness: the loss shall occur shortly, or within the same day as the event/disease happened or started. This period can be extended up to 5 days for people who do not regain consciousness after the incident (eg, illness/accident/suicide, etc). The families cannot enter this study later than 3 weeks after the death.

Potential participants with severe medical conditions for both bereaved partner and their children, such as serious physical impairment, intellectual deficit, severe child developmental problems, borderline personality disorder, history of psychosis (eg, schizophrenia, bipolar disorder), current substance use disorder (in the past 6 months), severe suicidal risk, or dementia will be excluded. Families receiving concurrent psychotherapeutic intervention for problems concerning loss and/or trauma will also be excluded.

### Recruitment and Randomization

A continuous recruitment will occur through referrals from crisis teams of the entire county of Hordaland in Norway. Health care units of the general hospitals in Hordaland, as for instance emergency and intensive care units, will also recruit for this study to secure the inclusion of those who lose a family member to acute disease.

Bereaved partners will be informed about the study the first time they are in contact with the health care personnel immediately after the death, and potential participants will be given a study information brochure. They will then receive a phone call to schedule an appointment for a first meeting at their homes (or at our center if they prefer so). At the end of this first meeting, the families will receive a sealed opaque envelope containing information regarding their random group allocation. Randomization will be previously performed by an external and independent research assistant who will use blocks of 6 families (3 allocated to each arm of the trial) [34]. For each block of 6, containing 3 intervention condition cards and 3 control condition cards, the researcher will randomly draw a card assigning it to one family according to its order of entrance in the study. This will help to randomly vary the order of interventions within each block. Due to the nature of the intervention, it will not be possible to blind participants to group assignment. However, the researchers who visit and phone the families have no previous knowledge concerning allocation until the end of the first meeting. In addition, they will not be involved in the intervention phase.

### Intervention

SEIB is a multidimensional clinical- and theory-based program designed to strengthen the resources of parentally bereaved children and their parents while adjusting to the major changes of a sudden and unexpected death of a parent/partner [26,27]. As suggested by others [28,30,35], SEIB attends to the unique needs of each bereaved family member, mapping the new ground that the families are entering and stimulating the benefits of the social network support. It has been acknowledged that bereavement is a process that differs among individuals; hence, intervention programs should be tailored to each individual’s needs [30].

Through a minimum of 5 sessions (or more if deemed necessary), SEIB’s focus is placed on: (1) stabilizing the situation and decreasing arousal (eg, by emotion regulation) [26,27], (2) facilitating individual coping skills and healthy grief reactions [26-28], (3) promoting positive parenting (warmth, open communication, effective discipline) [25,26], (4) encouraging the adaptive expression of emotions among family members [25,26], (5) empowering parent-child relationship and family interactions [25], (6) stimulating occupational and social functioning [26], and (7) activating/monitoring social support provision [27,28].

#### Session 1

Session 1 is to take place within the first 3 days after the loss, whenever possible (no later than 3 weeks post loss). The initial aim of this session is to reduce bodily activation by calming and stabilizing family members, and stimulating their perception of being cared for. In addition, this session focuses on parental psychoeducational information about their children needs’, stimulating open and direct communication, and ensuring that facts are shared within the family. In order to contextualize the facts, to establish coherence/structure, and secure equal access to facts within the family, every family member is invited to narrate their loss experience (facts, not feelings) with a special emphasis on the importance of hearing the children’s perspective. Furthermore, this session provides concrete advice on sleep, work/school, use of medication, participation in rituals, and how to maximize support from their social network (for both children and adults). At the end of this session, the parents receive a booklet as a guide for them to talk with their children about death [36].

#### Session 2

Session 2 takes place within 2 to 4 weeks post loss. The initial aim of this session is sharing of factual information concerning the circumstances of the death and the death notification, as well as new available information (ie, from police, health personnel, or others), ensuring that everyone in the family (including children) have access to the information they need. As in the first session, this session continues to secure open and direct communication within the family. In addition, psychoeducational information concerning basic and common grief reactions is combined with concrete advice for dealing with school/work re -entry. Grief and traumatic reminders, guilty feelings, and intrusive material are also addressed, and self-help methods introduced. The use of self-help methods is based on feedback from the family members on what they find particularly difficult. Usual relevant self-help methods refer to techniques that help to gain control over intrusive memories, reduce bodily arousal or tension, and improve sleep hygiene [37]. Finally, this session discusses how family members interact with each other, and how they can effectively support and take care of each other. New distribution of roles among family members and how to make good use of their social support networks are addressed.

#### Session 3

Session 3 takes place within 5 to 7 weeks post loss. The initial aim of this session is to address and process trauma aspects of the loss. Family members go through what they knew and thought about what happened, as well as their sensory experiences and body reactions at the time (especially the most difficult ones). If trauma-related problems persist, such as intrusive memories or thoughts, trauma focused methods such as Eye Movement Desensitization and Reprocessing (EMDR) [38] and Thought Field Therapy (TFT) [39] can be applied. The family members are advised to imagine having a conversation or write to the deceased and say goodbye (eg, writing a letter to the loved one, mentioning what they did not have time to say/do; ask for forgiveness if there are any regrets) [40]. A discussion around the balance between maintaining a constructive bond with the dead and keeping the lost one too close is also addressed. In addition, an emphasis is placed on living with grief over time, principally for the next coming months (eg, resume normal activities, interact with the environment, organize/tidying the dead person’s belongings, set goals for the family, use self-help techniques with videos found on the Internet). Finally, parents receive information for learning more about children’s reactions [41].

#### Session 4

Session 4 takes place within 3 to 6 months post loss. This session starts with a discussion on what has happened since the last meeting in order to: identify what is presently regarded as most important for the family and/or the individual family members; and reinforce the acquisition of new skills and the changes that were made as a family. A special emphasis is placed on living with grief over time and recovering daily functioning, particularly in the social and work/school spheres of life (eg, family interaction, be part of and sustain a helpful social network, etc). Additionally, if the dead continues to be kept very close (ie, nothing has been changed, as if the person died the day before), some changes are encouraged such as organizing/tiding up belongings, decreasing the number of grave visits and the amount of time spent in talking or thinking about the dead, and so on. The use of the “postponed worry-technique” [42] is introduced. Specific work regarding any sleep problems is included, as well as EMDR and TFT for trauma-related problems that persist over time. Finally, this session continues to stimulate family communication and discusses how they support each other.

#### Session 5

Session 5 takes place around or following the first anniversary of the death (12-13 months after death). This session starts with a discussion on how it is to have gone through a whole year without the lost person, and how was it to pass the anniversary in order to: identify what is presently regarded as most important for the family and/or the individual family members; reinforce the acquisition of new skills and the changes that were made as a family; and prepare for the life ahead. A special emphasis is placed on living with grief over time and recovering daily functioning, particularly in the social and work spheres of life. Finally, it is discussed how family members can give themselves permission to grieve less.

The rationale for both immediate-SEIB and delayed-SEIB versions relies on the need to minimize the initial misconceptions and maladaptive appraisals that may emerge in the aftermath of a traumatic event and that may exacerbate the onset of posttraumatic symptoms and complicated grief [27]. As a result, both immediate-SEIB and delayed-SEIB seek to enhance more appropriate coping responses that are expected to have an increasing effect over time. Also, early intervention may foster an open family climate that help the family to share facts and make decisions (eg, participating in rituals, school/work reentrance), favorable for them in a long-term perspective [27]. Traumatized parents tend to shield their children from facts, thus it seems important to stimulate proper levels of family communication, emotional expressiveness, and cohesion within the first weeks after a crisis event. This may decrease the individual and family arousal, increase family resilience, and stimulate a safer environment for trauma recovery [26]. These arguments constitute the base for our immediate-SEIB intervention and are in line with our clinical and research experience, showing bereaved people ask for immediate assistance [22,29]. The immediate-SEIB first follow-up period (session 4) is consistent with the International Classification of Diseases, suggesting that complicated grief meets its diagnostic criteria when the symptoms persist beyond 6 months after the death [43]. The immediate-SEIB second follow-up period (session 5) is consistent with the Diagnostic and Statistical Manual of Mental Disorders, requiring a minimum of 12 months [44].

The delayed-SEIB proposal is informed by the literature, given most preventive studies tend to start intervention within 2 to 6 months post loss [31,32]. For the delayed-SEIB intervention group, the first 3 sessions contain less advice on the acute handling of the situation (including children in rituals, return to work and school, securing social support) and less focus on reducing bodily activation by calming family members. They prioritize the traumatic aspects surrounding the death, and psychoeducation is adjusted to the reactions usually seen at this time-point (~3 months following the loss), when the unreality has abated and the social network is often less active in their support.

Four psychologists will be responsible for SEIB delivery. They have extensive clinical experience with grief, bereavement, and trauma, and are familiar with the theoretical and empirical background that lies beneath the development of SEIB. A SEIB manual was developed and reviewed together with the psychologists. The intervention protocol is described in session-by-session detail in order to maintain treatment fidelity. The psychologists will meet with the project leader and the manual developer (first and final author) during program implementation to secure supervision and adherence to the SEIB protocol. Session-by-session, short, semistructured logs in which the psychologists and family members note their own impressions about the intervention process will also be used and discussed in the supervision contacts.

### Outcome Measures

SEIB consists of a complex intervention [45], designed to tailor both individual and family needs, that is expected to entail several interacting components and outcomes. Considering the relevance of SEIB’s intervention components in the almost immediate aftermath of a potential traumatic loss, we anticipate the primary outcome of SEIB refers to traumatic stress symptoms of both parents/children. The secondary outcomes of SEIB may refer to the individual level of complicated grief, psychological wellbeing, daily functioning, and social support in parents and children, while tertiary outcomes possibly refer to the parenting/family level and include parental capacity, parenting practices, and family functioning.

Given SEIB's initial focus on the potential misconceptions/maladaptive appraisals that usually follow a sudden death, and the early provision of trauma-reducing self-help methods, it is foreseen that SEIB may reduce traumatic stress symptoms of both parents/children. The reduction of this symptomatology may favor psychological wellbeing and optimize daily functioning, which in turn may strengthen the individual sense of self-efficacy and the ability to use the support from others within the social network, minimizing the chances for complicated grief. For the surviving parent, these conditions may set the stage for a more resourceful, stable, and organized parent more considerate of the child’s needs. Enhanced emotional availability in parents, combined with SEIB’s family perspective, may ultimately improve family functioning. Textboxes 2 and Textboxes 3outline the parent and child self-report measures that will be used.

**The parent self-report measures.**

- Impact of Event Scale-Revised (IES-R) [46] – assesses the subjective distress following a traumatic event. It is composed of 22 items rated on a 5 -point scale ranging from ‘not at all’ (0) to ‘extremely’ (4).
- Inventory of Complicated Grief (ICG-19) [47] – assesses the severity of complicated grief symptoms. It is composed of 19 items rated on a 5 -point scale ranging from ‘never’ (0) to ‘always’ (4).
- General Health Questionnaire (GHQ-12) [48] – assesses general psychological wellbeing. It is composed of 12 items rated on a 4 -point scale. Items indicating health range from ‘more than usual’ (0) to ‘much less than usual’ (3), and items indicating illness range from ‘not at all’ (0) to ‘much more than usual’ (3).
- Work and Social Adjustment Scale (WSAS) [49] – assesses functional impairment at work, home, and social life. It is composed of 5 items rated on a 9 -point scale ranging from ‘not at all’ (0) to ‘very severely’ (8).
- Crisis Support Scale (CSS) [50] – assesses perceived and received support after the occurrence of a crisis event. It is composed of 7 items rated on a 7 -point scale ranging from ‘never’ (1) to ‘always’ (7).
- Parenting Coping Scale (PCS) [51] – assesses general parental ability to cope with the role of parenting. It is a brief single -item composed of 5 statements, forming a 5 -point scale rated from ‘coping very poorly’ (1) to ‘coping very well’ (5).
- Alabama Parenting Questionnaire (APQ) [52] – assesses several dimensions of parenting. A total of 14 items referring to parental involvement (7 items) and parental discipline (7 items) will be used and rated on a 5-point scale ranging from ‘never’(1) to ‘always’ (5).
- Family Assessment Device (FAD) [53] – assesses family climate and functioning. The General Functioning Scale is one of its subscales and the one that will be used. It focuses on family (un)healthy functioning and it is composed of 12 items rated on a 4-point scale ranging from ‘strongly disagree’ (1) to ‘strongly agree’ (4).

**: The child self-report measures**

- Children’s Impact of Event Scale (CRIES-8) [54] – assesses the subjective distress following a traumatic event. It is composed of 8 items rated on a 4-point scale ranging from ‘not at all’ (0) to ‘often’ (5).
- Inventory of Prolonged Grief for Adolescents (IPG-A) [55] – assesses symptoms of prolonged grief disorder. It is composed of 30 items rated on a 3-point scale ranging from ‘almost never’ (1) to ‘always’ (3).
- Strengths and Difficulties Questionnaire (SDQ) [56] – assesses psychological adjustment in children and adolescents. It is composed of 25 items rated in a 3-point scale ranging from ‘not true’ (0) to ‘certainly true’ (2), and an impact supplement focusing on functional impairment.
- Alabama Parenting Questionnaire (APQ) [52] – encloses a parent and child version. The equivalent items of the parent version will be used in the child version.
- Family Assessment Device (FAD) [53] – can be filled in from 12 -years -old onward. The same items will be used for both parent and child.

Measures were selected based on relevance, satisfying psychometric properties, brevity, and availability in the Norwegian language.

To assess both children’s and parents’ perspectives on the help and support they received following the death, we developed a self -report measure to relate their perception of SEIB and/or care as usual throughout their participation (Help Questionnaire).

As shown in Textbox 4, the families will be assessed at 4 time-points: baseline (T1), and after 3- (T2), 6- (T3), and 9-months (T4) follow-up. T1 will just comprise the use of two questionnaires: GHQ-12/SDQ and IES-R/CRIES-8. Given the closeness in time of T1 to the death, we expect the families to be acutely distressed at this time. Apart from T1, all the above-mentioned parent and child questionnaires will be included at the other time-points for assessment. These questionnaires will provide useful information on future power calculations and possible SEIB outcomes.

**Assessment time-points and measures for all participants.**

T1: Baseline

Sociodemographics

- Parent: IES-R; GHQ-12
- Child: CRIES-8; SDQ

T2: 3 months

Help Questionnaire

- Parent: IES-R; ICG-19; GHQ-12; WSAS; CSS; PCS; APQ; FAD
- Child: CRIES-8; IPG-A; SDQ; APQ; FAD

T3: 6 months

Same as T2

T4: 9 months

Same as T2

We will inspect data on the flow of participants throughout the trial in order to estimate recruitment and final retention rates. Partial retention rates for those entering the trial who do not complete questionnaires at 9-months follow-up will also be calculated. Besides, the psychologists will inform the research team about the number of attended sessions and reasons for dropping out treatment.

To assess recruitment materials and procedures we developed a brief questionnaire that will be sent to the recruiting agencies at the end of the recruitment phase. The usefulness of randomization and data collection materials (eg, sealed opaque envelopes, informed consent forms, questionnaires) will be evaluated by the researcher who will fill in a ‘brief family log’ after visiting the family.

### Sample Size and Statistical Analyzes

In light of the small state of knowledge about early intervention in the aftermath of a potential traumatic death of a partner/parent, this study is not aimed to test SEIB effectiveness. Power analysis is not used to determine the final sample size, because it is not recommended for pilot studies that do not rely on inferential statistical tests [57]. Following a general rule of thumb that suggests the inclusion of 30 participants or more to determine a parameter (eg, mean/standard deviation) of an outcome variable [58], we will recruit a total of 60 families (30 families in each group). Rather than focusing on hypothesis testing, this pilot study builds on the practical limitations of recruitment and topics of incertitude, such as the need to gather initial estimations for sample size calculation [57,59,60].

Statistical analyzes will be computed using IBM SPSS Statistics and multiple imputation methods will be used to impute values of missing data [61]. Descriptive data will be computed to characterize the sample. On the basis that pilot study analyzes should be mostly descriptive and should provide confidence interval estimation [60,62], descriptive statistics (including mean, percent, standard deviation, and range), and confidence intervals will be used to describe all outcome variables at T1, T2, T3, and T4 providing important information regarding recruitment and randomization procedures, data collection forms, retention rates, future power calculations, and SEIB most appropriate outcomes.

Given the acknowledged low power of pilot studies [57], an emphasis will be placed on evaluating indices of clinical significance. Independent of the normal distribution and sample size, the effect size estimation has been considered a strong predictor of clinically meaningful change in studies with small sample sizes [63]. Accordingly, Cohen’s *d* effect size of the differences in outcomes between groups and Cohen’s *d* effect size of change from baseline to follow-up will be performed and judged as follows: small (*d* ≥ 0.2), moderate (*d* ≥ 0.5), large (*d* ≥ 0.8), or very large (*d* ≥ 1.3) [64]. The preliminary early effects of SEIB will be addressed by comparing descriptive data and effect sizes at T2 of both immediate-SEIB group and delayed-SEIB group (receiving care as usual at T2). The preliminary effects of both immediate and delayed SEIB versions will be addressed by comparing descriptive data and effect sizes of the immediate-SEIB group at T2 and T3 to the delayed-SEIB group at T3 and T4.

### Ethical Considerations

The Regional Committee of Research and Ethics in Western Norway (no. 599832) approved the design and procedures of this study. The ethical principles for research in the social sciences and the humanities will follow the Helsinki Declaration guidelines' [65]. From the start, participants’ needs will prevail over research interests. The agreement to participate in the study and an explicit written informed consent will precede data collection. Participants will be informed about all the relevant aspects of the study (eg, aims, methods, allocation, etc). They will also be reassured that their participation is voluntary and that they can withdraw at any time without detriment. The participant’s privacy and confidentiality will be assured throughout all research phases. Both researchers and psychologists will monitor potential adverse effects or special circumstances that will require participants’ removal from SEIB. Additionally, all participants can make use of care as usual, which seems particularly significant to those in the control condition who are waiting for the delayed SEIB program.

The participant’s informed consent will be obtained shortly after the death. However, several researchers have underlined the difference between being distressed, and not being capable of making decisions around enrollment in a bereavement research study [66,67]. In fact, bereaved people are often involved in complex decision-makings concerning the funeral planning, their financial situation, and so on. Besides, they do not report their research participation as undue strain [68]. Acknowledging a temporary distress, they emphasize their research participation as positive, without showing regrets, and highlight the benefits of participation when they are being respectfully, sensitively and properly cared for [68]. Nevertheless, given the closeness of T1 to the death, where the families may be acute distressed/disturbed, it is necessary to limit the potential strain that can be added to the families. Presenting many questions to the family members at this early time following the loss may lead to problems or even discourage their retention in the study. In addition, their diminished cognitive resources may negatively affect their ability to properly fill in many questionnaires at this time. Therefore, T1 will just comprise the use of the above -mentioned 2 questionnaires.

### Project Organization

This is a one-center study conducted in Norway for a timeframe of 3 years. It was initiated in November 2014 and will be completed by November 2017. An expert advisory board with representatives from (inter)national universities and institutions with special competencies in the grief/trauma field will provide on the study during the research process. This is of primary importance as there are few international (and none Norwegian) studies informing early follow -up practices following loss and trauma.

## Results

The present pilot study is ongoing and started enrolment of families in August 2015. Data collection is expected to be completed in June 2017.

## Discussion

### Implications of the Study

This pilot study will evaluate the plausibility and practicability of a future main trial. Furthermore, it will explore the preliminary short -term effects of an early multidimensional program designed to promote families’ grieving process and adjustment after a sudden death of a partner/parent, and to decrease the risk for complicated grief in both bereaved children and their surviving parents. The rationale for this study is embedded in the existing literature [26-28], our clinical experience, and the wishes for immediate assistance, outreach help, and help for their children expressed by the bereaved themselves [22,29].

### Strengths and Limitations

To our knowledge, this is the first study proposing an early preventive program that starts up in the first days following loss with the potential to adapt the intervention to the needs of bereaved families over time. It is also one of the first trials employing the delayed intervention design in the grief field in order to explore the effect of time on outcome variables. Therefore, this study will provide preliminary knowledge about the value of very early intervention following bereavement, which crucially calls for empirical support and clarification. In addition, the delayed intervention design overcomes some important ethical, methodological, and logistical constraints, because it assures intervention for all participants [69]. On the one hand, it seems to suit the needs of the families, who may show a strong wish for immediate assistance [22,29]. On the other hand, it can minimize possible dropout effects as our former experience in conducting research makes us believe that these families are likely to show a high expectation of receiving intervention from us. Accordingly, the delayed intervention design minimizes possible disappointment effects of those who are not allocated to their treatment choice, preventing the risk of refusing randomization [70]. As a result, we consider that the use of the delayed intervention design will maximize recruitment and retention. Consequently, considering that the lack of evidence of preventive interventions for bereaved has been linked to higher levels of complexity in implementing these studies [30], we think this delayed intervention design takes into account the interests of our bereaved families, promoting the study’s integrity, its effective implementation, and full potential.

Nonetheless, this pilot study may entail a number of possible limitations. First, recruitment constraints may lead to a small and/or possibly selective sample. The Norwegian population is small and our recruitment potential is not large, given that we need to wait for the potential traumatic event to happen. Besides, the intervention takes place at our center, which means we can only recruit in the surrounding county. Further, the bereaved families are contacted at the first time they meet health care personnel following the death, which may be particular demanding for some families, undermining their willingness to participate in the study.

Second, both early-SEIB and delayed-SEIB intervention groups can make use of care as usual, which may lead to carry -over effects [71]. This refers to the first support and help these families can receive from the crisis team, general practitioner, hospital, church, and/or school in the community. In communities outside the urban area, and in recruiting hospital wards, care as usual will vary greatly. However, families receiving other concurrent psychotherapeutic intervention will be excluded from the study and the Help Questionnaire may help to monitor some of these potential carry -over effects.

Third, a longer follow -up assessment should have been considered, as it would allow us to report on possible long -term effects, and to explore the sustainability of change over time [72]. Financial unfeasibility is the main reason that hinders the likelihood of looking at possible SEIB's long -term effects. Grant providers should recognize bereaved people as a particularly vulnerable, sensitive, and 'hard -to -reach' group that requires additional resources, both financial and from the research team.

Fourth, family participation may entail a small burden because it involves the fulfillment of a set of questionnaires at 4 time-points. The expected distress in relation to filling in questionnaires is anticipated to be highest at T1, because it takes place in the near aftermath of the death where families may be stunned or shocked. We try to minimize this burden by using 2 questionnaires at T1 (with an estimate short time to complete them: 15 to maximum 30 minutes), and having a researcher present with the families to provide help and assistance with the task. Besides, previous research have showed that the burden of filling in questionnaires was outweighed by the meaningfulness of participation, as it tends to increase families’ understanding of their grief reactions [68]. The intervention will take 1 to 2 hours per session and will require that the families express what help they think they need. Considering the needs of suddenly bereaved families, SEIB will hopefully operate as a protective factor, promoting individual and family’s adjustment. This is likely to stimulate a safer and less stressful mourning environment, which may even help to prevent further maladaptive trajectories. Therefore, we consider that the benefits for the individual and the family exceed the minimal constraints that this study may entail.

### Conclusion

In sum, rather than evaluating SEIB efficacy or effectiveness, the current pilot study assesses the feasibility of a fully-powered trial. This study encompasses 3 major strengths that are the development of SEIB, the sample of bereaved families at considerable risk for complicated grief, and the use of the RCT with a delayed intervention design. It will possibly expand knowledge on the usefulness of a proactive, holistic, and supportive approach that may be of major importance for public mental health services, governments, and crisis teams.

## Abbreviations

APQ: Alabama parenting questionnaire
CRIES-8: children’s impact of event scale
CSS: crisis support scale
EMDR: eye movement desensitization and reprocessing
FAD: family assessment device
GHQ-12: general health questionnaire
ICG-19: inventory of complicated grief
IES-R: impact of event scale-revised
IPG-A: inventory of prolonged grief for adolescents
PCS: parenting coping scale
PTSD: posttraumatic stress disorder
SDQ: strengths and difficulties questionnaire
SEIB: systematic early intervention for bereaved
T1: baseline
T2: after 3-months follow-up
T3: after 6-months follow-up
T4: after 9-months follow-up
TFT: thought field therapy
WSAS: work and social adjustment scale
